# Weakly supervised learning and interpretability for endometrial whole slide image diagnosis

**DOI:** 10.1177/15353702221126560

**Published:** 2022-10-25

**Authors:** Mahnaz Mohammadi, Jessica Cooper, Ognjen Arandelović, Christina Fell, David Morrison, Sheeba Syed, Prakash Konanahalli, Sarah Bell, Gareth Bryson, David J Harrison, David Harris-Birtill

**Affiliations:** 1 School of Computer Science, University of St Andrews, St Andrews KY16 9SX, UK; 2 Department of Pathology, Queen Elizabeth University Hospital, Glasgow G51 4TF, UK; 3 School of Medicine, University of St Andrews, St Andrews KY16 9TF, UK

**Keywords:** Digital pathology, weak supervision, adenocarcinoma, hyperplasia, endometrial cancer, cancer detection, iCAIRD, XAI, interpretable AI

## Abstract

Fully supervised learning for whole slide image–based diagnostic tasks in histopathology is problematic due to the requirement for costly and time-consuming manual annotation by experts. Weakly supervised learning that utilizes only slide-level labels during training is becoming more widespread as it relieves this burden, but has not yet been applied to endometrial whole slide images, in iSyntax format. In this work, we apply a weakly supervised learning algorithm to a real-world dataset of this type for the first time, with over 85% validation accuracy and over 87% test accuracy. We then employ interpretability methods including attention heatmapping, feature visualization, and a novel end-to-end saliency-mapping approach to identify distinct morphologies learned by the model and build an understanding of its behavior. These interpretability methods, alongside consultation with expert pathologists, allow us to make comparisons between machine-learned knowledge and consensus in the field. This work contributes to the state of the art by demonstrating a robust practical application of weakly supervised learning on a real-world digital pathology dataset and shows the importance of fine-grained interpretability to support understanding and evaluation of model performance in this high-stakes use case.

## Impact Statement

Our article contributes to the growing body of work on weakly supervised deep learning for diagnostic tasks in digital pathology, a promising field of enquiry which has potential to significantly benefit patients with faster and more accurate diagnoses while relieving pathologists from the burden of time-consuming manual annotation necessary for fully supervised methods. We use real-world endometrial dataset collected from multiple labs and demonstrate over 85% diagnostic accuracy despite huge variation in image size, magnification, and staining across the data. We show in detail how we achieved these results to enable replication of our work. We also apply three interpretability methods to our trained model, and in consultation with expert pathologists demonstrate that weakly supervised learning can produce models that learn to identify highly salient features in the data. Work of this kind is crucial to support widespread, and crucially, safe adoption of these powerful deep-learning techniques in digital pathology.

## Introduction

Digital pathology is a subfield of pathology that focuses on acquisition, management, sharing, and interpretation of pathology information from digitized specimen slides. It can be traced back to the 1960s when first telepathology experiments took place.^1^ Since the idea of virtual microscopy appeared in several areas of life science research in the 1990s,^2^ digital imaging has revolutionized the practice of pathology in many ways – not only decreasing costs associated with storage and handling of glass slides, but also shortening waiting times on diagnostic decisions, increasing precision of diagnosis, and enabling faster treatment.

Compounded by demographic changes, pathology services in healthcare systems across the world are under pressure from increasing demand and resource limitations – even as the digitization of specimen samples paves the way for the use of computational image analysis technology and software. Modern artificial intelligence (AI)-based systems have the potential to handle vast amounts of data, far in excess of what humans are capable of, and as such have huge potential to assist pathologists in their diagnostic work and allay these pressures.

Endometrial cancer is the most common gynecological cancer in industrialized countries. It is the fourth most common cancer,^3^ with around 9700 new cases in the UK every year – fortunately, deaths from this type of cancer are decreasing year on year due to early diagnosis via transvaginal ultrasound or biopsy tests. Deep learning can assist pathologists in automating the image analysis process and thereby speed up life-saving detection, and so increasingly AI solutions are applied to endometrial whole slide images (WSIs) to detect cancers and segment regions of interest.

However, training deep-learning models on gigapixel size WSIs is prohibitively computationally expensive, and so patching approaches (in which the input image is divided into a series of small patches before training) are typically employed in this use case. Another constraint associated with machine learning on WSIs is the high cost of label acquisition – in order to perform supervised learning, many slides are necessary, and each slide requires time-consuming manual annotation by experts (in comparison with image classification or segmentation tasks on natural image data, which may be easily labeled by laymen). This cost can be prohibitive at scale, and so it is often necessary to use weakly supervised training methods, as we employ herein.

Weakly supervised learning methods are often used to train deep neural networks to detect and localize different cancer types, but understanding how these models work can be difficult due to the opaque nature of their “black-box” architecture. This is a broader problem in deep learning as a whole, but particularly relevant here due to the high-stakes use case. If our model is performing well because of some spurious correlation in the data, we definitely want to know about it before using that model for clinical diagnostic tasks. This concern has been a topic of much study, and various approaches to model interpretability for machine learning in medicine have been developed.^4^

Multiple instance learning (MIL) is a variation of supervised learning that assigns a single class label to a set of instances (e.g. the patches extracted from of a WSI).^5^ The original MIL algorithm restricts its scope to binary classification problems, based on the assumption that if at least one patch belongs to the positive class, then the entire slide should be classified as positive, whereas a slide should be classified as negative if all patches are of the negative class. This assumption is reflected in the rigid, non-trainable aggregation function of max pooling which simply uses the patch with the highest predicted probability for the positive class for the slide-level prediction, rendering MIL in this form suitable for neither multiclass classification nor binary classification problems in which no intrinsic positive/negative assumption can be made.^6^ Further work has employed different aggregation methods to provide better slide-level accuracies. For example, attention-based deep MIL^7^ replaces permutation-invariant aggregation operators (e.g. the maximum, or mean) with a trainable weighted average using a two-layered neural network, corresponding to the attention mechanism.

Other learning-based aggregation methods include that of Campanella *et al*.,^8^ which employs a full inference pass through the dataset to rank the patches according to their probability of being positive, and learning takes place only on the top-ranking patches of each slide. The aggregation of patch-level to slide-level classification is carried out by a recurrent neural network (RNN), such that the most suspicious patches in each slide are sequentially passed to the RNN to predict the final slide-level classification.

The weakly supervised approach for classification of whole slide lung cancer images proposed by Wang *et al*.^9^ takes advantage of a patch-based fully convolutional network for discriminative block retrieval. Furthermore, context-aware feature selection and aggregation strategies are proposed to generate globally holistic WSI descriptors.

The clustering-constrained attention MIL^10^ requires only slide-level labels while being data-efficient, adaptable, and capable of handling multiclass subtyping problems. This method has been applied to a variety of datasets such as Camelyon 16, Camelyon 17, and The Cancer Genome Atlas (TGCA) datasets.

Huang and Chung^11^ propose a weakly supervised convolutional neural network (CNN) method for localizing cancerous evidence on histopathology images. Unlike the conventional feature-based approaches, the proposed Cancerous Evidence Localization Network (CELNet) does not rely on specific feature descriptors but learns discriminative features for localization from the data. The localization results based on cancerous evidence localization map highlight the localized evidence which provides visual assistance for the pathologists.

CNN-based models used for endometrial cancer detection along with visualization techniques have provided pathologists better interpretability of model diagnoses. While state-of-the-art methods for WSI diagnosis rely on fully supervised methods, experimental results of weakly supervised methods as discussed above demonstrate competitive performance and can offer a realistic solution for triage where acquiring detailed annotations is impractical.

The computer-aided diagnosis (CADx)^12^ approach uses a convolutional neural network and attention mechanisms for detecting four fine-grained classes of endometrial tissue, namely, the (normal) endometrium within a regular menstrual cycle, endometrial polyp, endometrial hyperplasia, and endometrial adenocarcinoma. The hematoxylin and eosin–stained (H&E) slides used in this experiment were scanned at 10× or 20× magnification. Interpretability is provided by highlighting the histopathological correlations of local (pixel-level) image features to morphological characteristics of endometrial tissue.

Hong *et al*.^13^ employ a customized multiresolution deep convolutional neural network that predicts molecular subtypes and 18 common gene mutations in endometrial cancer based on digitized H&E-stained pathological images. The models learn human interpretable and generalizable features, indicating potential clinical application without sequencing analysis.

Li *et al*.^14^ propose a weakly supervised framework that learns the feature representations of various lesion areas from endometrial histopathology WSIs. The proposed framework consists of a contrastive learning network as the backbone and a designed contrastive dynamic clustering (CDC) module to embedding the lesion information into feature representations.

However, all the above approaches are constrained to binary classification, which significantly limits their application to real-world datasets. For this reason, we here adopt the clustering-constrained attention multiple instance learning (CLAM) method as proposed by Lu *et al*.,^10^ which resolves these issues by extending the MIL paradigm to support multiclass subtyping problems.

In addition, the use of attention-based aggregation allows for straightforward attention heatmap generation, which is useful for the evaluation of model behavior.

## Materials and methods

In this article, we adopt the weakly supervised CLAM method proposed by Lu *et al*.^10^ for slide-level diagnosis of endometrial H&E WSIs. This task is framed as a multiclass classification problem, with three classes: malignant, benign, and insufficient.

We integrated the CLAM method with Philips Pathology Software Development Kit (SDK) to enable its use with endometrial WSIs in iSyntax format. Code to achieve this is available at https://github.com/Mahnaz54/icaird-weaklysupervisedlearning, which we believe will prove useful to the interested community as the CLAM method has not previously been applied to iSyntax format data. Moreover, we extend this approach using end-to-end saliency-based segmentation to identify key regions within patches at the pixel level, thereby generating far more detailed segmentation maps for interpretability purposes than attention mapping can offer from only weakly labeled data. We also use feature visualization via input optimization to identify distinct morphologies learned by the model to differentiate between tissue classes and discuss how these interpretability methods enable us to better understand the trained model behavior with expert pathologist input.

### Data collection

The H&E WSIs used to train, evaluate, and test the algorithm in this article are part of the national database for automated reporting of the digital diagnostics within the pathology AI stream of iCAIRD (Industrial Centre for Artificial Intelligence Research in Digital Diagnostics), used to develop a system to identify and segment pathologies in endometrial H&E WSIs. These slides are of different sizes and scanned at different resolution levels, and also vary in color, as H&E staining intensity and hue vary across different labs. No color normalization has been applied to the slides or patches at any step of the pipeline, to ensure that our trained model will generalize to unseen hue and intensity in the test set, and each slide is from a different patient to ensure robustness to interpatient application of the trained model.

**Figure 1.**
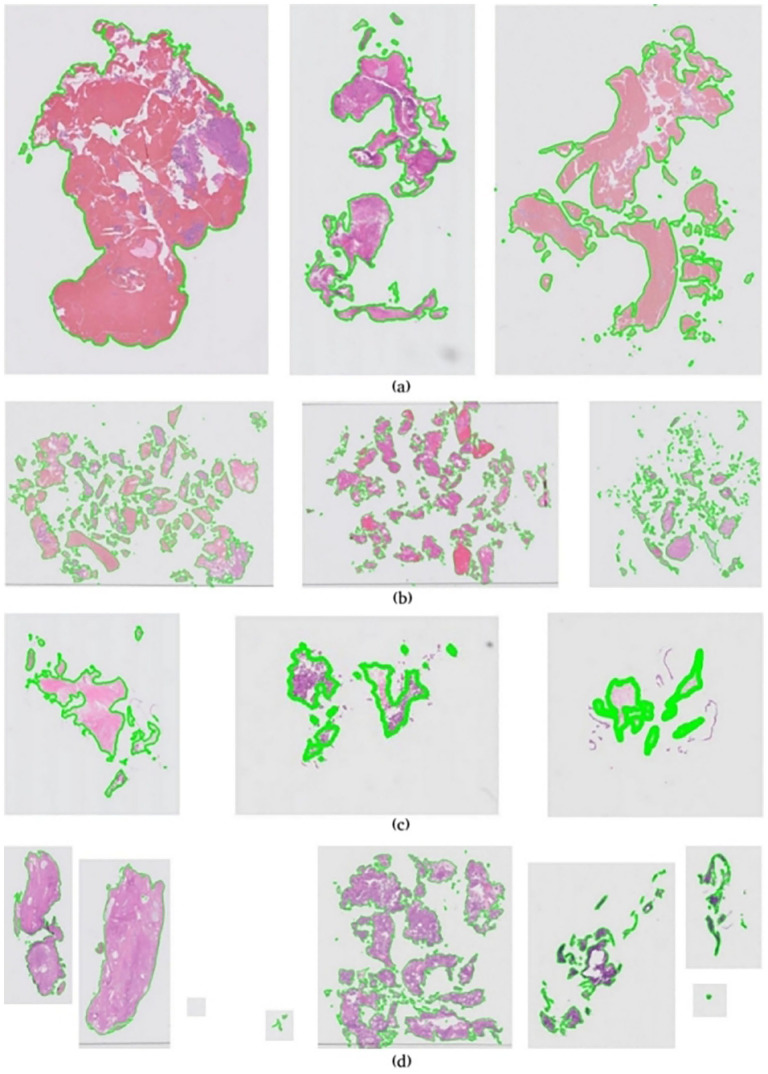
Overview of data complexity in endometrial dataset. (a) Color variations in endometrial whole slide images. (b) Intense fragmentation in endometrial whole slide images. (c) Low-resolution scanned whole slide images. (d) Multi subimages in endometrial whole slide images. (A color version of this figure is available in the online journal.)

Due to these factors, our dataset exhibits a wide variance – Figure 1 illustrates an overview of data complexity in this experiment. Figure 1(a) to (d) shows examples of color variations, fragmentation intensity, slides scanned at low resolution, and few slides that contain more than one subimage, respectively.

The tissue blocks for this study originate from Glasgow Royal Infirmary (NG), Southern General Hospital (SG), Royal Alexandria Hospital (RAH), and Queen Elizabeth University Hospital (QEUH) (all in Glasgow, Scotland), each with independent tissue handling including fixation and tissue processing. New tissue sections were cut from the tissue blocks at one of two different thicknesses (3 or 4 µm) and stained with one of four different H&E protocols (routine H&E, muscle biopsy protocol, neuro protocol, and paeds protocol). Together, these options maximize WSI variance and thereby decrease the likelihood of overfit to any one lab. Table 1 gives an overview of the main categories (slide-level labels) and subcategories, along with the numbers of samples in each main category for train, validation, and test sets. As the table illustrates, the dataset is quite imbalanced. For this experiment, only the whole slide-level diagnosis (slide-level labels) has been used, and no segmentation annotations or subcategory labels are used for training.

**Table 1.** Number of samples in each category for iCAIRD endometrial dataset.

Category	Subcategory	Train	Validation	Test
Malignant	- Hyperplasia with atypia - Adenocarcinoma - Carcinosarcoma - Sarcoma	444	148	269
Other/Benign	- Hormonal Change - Inactive/atrophic - Menstrual - Secretory - Proliferative	954	318	595
Insufficient	- Insufficient	99	33	47

We received a total of 2910 WSIs of endometrial biopsies with just one WSI per patient. All WSIs from two of the staining labs (staining labs 6 and 8) and 10% of WSIs from other labs which were randomly selected and balanced over the category, subcategory, and staining labs were set aside as test set. Remaining slides were used for training. We selected 25% of the training slides randomly but balanced over category, subcategory, and staining labs for validating the algorithm during the training. This resulted in 1497 train, 499 validation, and 911 test samples as shown in Table 1.

Overall distribution of data between train, validation, and test sets based on staining sites and staining labs is shown in Tables 2 and 3. To assess the generalization ability of our model to unseen staining protocols, the entirety of the data from two of the labs was withheld from the training set and included only in the test set.

**Table 2.** iCAIRD endometrial dataset distribution over staining sites.

Staining Site	Train	Validation	Test
NG	367	140	253
SG	407	128	277
RAH	336	100	164
QEUH	387	131	217
Total	1497	499	911

NG: Glasgow Royal Infirmary; SG: Southern General Hospital; RAH: Royal Alexandria Hospital; QEUH: Queen Elizabeth University Hospital.

**Table 3.** iCAIRD endometrial dataset distribution over staining labs.

Staining Site	Train	Validation	Test
Lab-1	268	75	37
Lab-2	260	85	38
Lab-3	241	90	37
Lab-4	263	83	37
Lab-5	236	83	34
Lab-6	–	–	343
Lab-7	229	83	34
Lab-8	–	–	351
Total	1497	499	911

The endometrial H&E WSIs used in this experiment are of iSyntax format. The iSyntax image format combines the medical-grade image quality of JPEG 2000 with the speed of JPEG and enables scalable cost-effective image storage both on premise and in the cloud, which makes it a distinguished image format for storing pathology WSI. The wavelet transformation technology allows users to zoom and pan through WSI images quickly. iSyntax encoding and decoding can be processed in real-time, and because the wavelet technology obviates the need for the redundant storage of lower magnification images in a pyramid format, it results in a 25% smaller file size. Unlike other formats that have a limited dynamic range, the iSyntax pathology format allows for medical image quality featured by arbitrarily high bit-depths, unlimited number of channels, lossless and lossy compression, and progressive decompression in terms of resolution and quality.^15^

### Annotations

The annotation process stratified each slide into four primary diagnostic categories: Endometrial Carcinoma, Carcinosarcoma, Uterine Sarcoma, and Endometrial Hyperplasia with Atypia. All slides from a balanced set of benign and malignant endometrial biopsies were digitally scanned on a Philips UFS Scanner as iSyntax WSIs. These were exported from the Philips Information management System and converted to OME-Tiffs using Glencoe Software as the original iSyntax images were not compatible with QuPath (Version v0.2.3). Images were manually annotated on a touchscreen Microsoft Surface Studio using a Surface Pen. The annotation protocol was to define the overall slide class followed by manual annotation of any different classes present on the WSI. Annotation classes were labeled in QuPath and annotations and labels were exported. The annotation vector files were aligned to the original iSyntax images for analysis. The annotation process was tracked in a bespoke Microsoft Access Database.

Each slide was randomly assigned to one of four participating Consultant Pathologists for annotation. Each of the participating pathologists had a subspecialist interest in Gynecological Pathology and participated in the National Gynecological Pathology External Quality Assurance Scheme. Primary annotation was performed either by one of the four pathologists or by a Biomedical Scientist, who were specifically trained for this project. All annotations done by a Biomedical Scientist were signed off by one of the study pathologists.

These annotated slides were divided into three sets: a training set, a validation set, and a test set, none of which overlap. Besides annotations on the digital slides themselves (detailed annotations/subcategories), the pathologists have supplied the diagnosis decision (main categories/slide-level labels). In this experiment, we only use the diagnosis decisions. The category labels are defined as follows:

1. Malignant: If a slide contains any of the four primary diagnostic categories (Endometrial Carcinoma, Carcinosarcoma, Uterine Sarcoma, and Endometrial Hyperplasia with Atypia), it is labeled as Malignant at slide level.
2. Insufficient: A slide which does not contain enough tissue on it to make diagnosis is labeled as Insufficient. Often an insufficient slide contains a tiny amount of tissue surrounded by blood and mucus.
3. Other/Benign: Slides with Menstrual/Shedding Endometrium, Inactive/Atrophic Endometrium, Hormonal Change, Proliferative Endometrium, and Secretory Endometrium are categorized as Normal/Benign.

### Weakly supervised whole slide analysis pipeline

**Figure 2.**
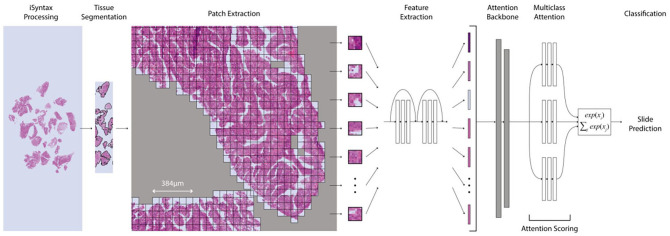
Weakly supervised WSI classification from patch features. (A color version of this figure is available in the online journal.)

The deep-learning-based weakly supervised method used in this article is a CLAM method proposed by Lu *et al*., which we integrated with the Philips Pathology SDK to enable the processing of iSyntax WSIs. CLAM is a deep-learning-based weakly supervised method that uses attention-based learning to automatically identify subregions of high diagnostic value to accurately classify the whole slide while also utilizing instance-level clustering over the representative regions identified to constrain and refine the feature space. For whole slide-level learning without annotation, CLAM uses an attention-based pooling function for aggregating patch-level features into slide-level representations for classification. Figure 2 shows the structure of the pipeline for weakly supervised whole slide analysis.

To avoid repetition, we just give a brief description of different steps of the pipeline in this article. Details for each step can be found in the paper by Lu *et al*.^10^

#### Tissue segmentation and patching of the WSI

The WSIs are encoded in a pyramid structure consisting of multiple images at different resolutions. The baseline image has the highest resolution. WSIs generated at diagnostic resolution are exceptionally large: a typical WSI may contain 100,000 × 100,000 pixels. The large size of WSIs makes it necessary to break down the WSIs to smaller patches for analysis. For each WSI, the pipeline reads in the slide to the memory at a downsampled resolution and then segments the tissue from the background to reduce the irrelevant white space in the slide. The segmentation process starts with converting the downsampled WSI from RGB to HSV color space. A binary mask is then created after applying median blurring to smooth the edges and thresholding the saturation channel of the smoothed image. The small gaps and holes will be filled by morphological closing. Finally, the deleted foreground contours are filtered based on an area threshold and the coordinates of the contours are saved. A segmentation mask for each WSI is then created for visual inspection. Patches are cropped from the segmented tissue area at the specified magnification (level 0) at the desired patch size (256 × 256). The coordinates of the patches are saved to avoid storing the patches in memory. The number of patches extracted for each slide depends on the size of the slide and the magnification level. Patches also can be extracted with overlap, but in this experiment, we have not used overlapping patches.

#### Feature extraction

Features are then extracted from each patch using a deep neural network – in our case, a ResNet50 model pretrained on ImageNet. The pretrained ResNet50 model is modified by adding an adaptive mean-spatial pooling after the third residual block of the network to convert each patch into a 1024-dimensional feature vector. Feeding these extracted features as inputs to a deep neural network results in faster training time and lower computational cost, which makes training a deep-learning model on substantial number of slides practical.

#### Instance-level clustering and attention scoring

The feature vectors are then passed to the rest of the pipeline to be clustered. For each class, the attention network ranks each patch in the slide and assigns an attention score based on its relative importance to the slide-level diagnosis. Attention-pooling weighs patches by their respective attention scores and summarizes patch-level features into slide-level representations, which are used to make the final diagnostic prediction. These attention scores also can be visualized as a heatmap to identify which regions the model used for diagnosis. The regions of high attention are displayed in red (positive evidence, high contribution to model’s prediction relatively to other patches) and the regions of low attention are displayed in blue (negative evidence, low contribution to model’s prediction relatively to other patches).

### Saliency segmentation

One of the largest problems in machine learning from WSIs is the lack of available pixel-level annotations for training. Classifying intraslide regions requires many hours of expert annotation, which is often prohibitively costly and time-consuming – hence the weakly supervised approach we adopt.

A common way of obtaining class-specific segmentation in this context, without the need for human annotators, is by inspecting the attention apportioned to each patch by the attention backbone component of our classifier model, as described above. This approach is useful for validating model predictions, as it enables easy inspection of smaller regions and insight into which areas were more or less instrumental in producing the final slide prediction – however, it can tell us little about smaller learned features present within patches, other than whether they are present or not, and produces segmentations too coarse for clinical use. To create more detailed saliency maps to aid in understanding and validating our model’s behavior, we here outline an end-to-end saliency-mapping algorithm that generates pixel-level segmentations for each patch.

For this, we use Hierarchical Perturbation (HiPe),^16^ a saliency-mapping method which is both model-agnostic and highly computationally efficient – this is necessary in order to mitigate the huge computational cost of pixel-level attribution on gigapixel input images. Given that our model is dual-stage, combining first feature extraction followed by a classification network, and that in this work we tested different model architectures, patch sizes, and other hyperparameters, model agnosticism was also key in this choice of saliency algorithm.

As our goal is to segment tissue, as well as to generate saliency maps for interpretability purposes, in this work we adapt HiPe to focus on perturbing regions not with higher saliency, but with lower saliency variance between classes – thus, we optimize for more detailed mapping in regions where the model is less certain which class is predominant by adapting HiPe to operate over all classes simultaneously and replacing the standard threshold function:

$$t\left(s,m\right)=\{\begin{array}{ll} 1 & if\;max\left(s\;{}^{\circ}\;m\right)\geq min\;s+\frac{\left(max\;s-min\;s\right)}{2}\\ 0 & otherwise \end{array}$$

with the following:

$$t\left(s,m\right)=\{\begin{array}{ll} 1 & if\;max\left(V\left(s_{i}\;{}^{\circ}\;m_{i},\;for\;i=0,\;.\;.\;.\;,C\right)\right)\leq V\left(min\;s_{i}+\frac{max\;s_{i}-min\;s_{i}}{2},\;for\;i=0,\;.\;.\;.\;,C\right)\\ 0 & otherwise \end{array}$$

where $V(X)=\frac{1}{n}\sum_{i=0}^{n}{\left(x_{i}-x\right)}^{2}$ , and *C* denotes the number of classes (in this case, three: malignant, insufficient, and benign/other). This results in fine-grained segmentation maps that can be compared with the ground truth labels provided by the experts.

### Feature visualization

Interpretability of deep neural networks can be helpful to healthcare. Feature visualization is one the fundamental building blocks that combined with additional tools helps to see what a network is looking for by generating examples.

Neural networks, in general, are differentiable with respect to their inputs. Iteratively tweaking the input toward a specific goal using the derivatives can help find out what kind of input causes that certain behavior. Different optimization objectives show what different parts of a network are looking for. To create examples of output classes from a classifier, we can optimize the class logits before the softmax or optimize the class probabilities after the softmax.

As proposed by Erhan *et al*.,^17^ learned features for each class (class logits before the softmax) can be visualized via input optimization in order to better understand how a trained model discriminates between classes. Hence, to visualize what the model is using to discriminate between the classes, we optimize the input image using derivatives for each class to maximize the output of the model for that image.

## Results

The CLAM model described in section “Materials and methods” was trained on training endometrial WSIs of three different classes (Malignant, Insufficient, and Other/Benign). It was then evaluated on validation set and tested on test set WSIs. We also trained multiclass variant of the MIL model proposed by proposed by Lu *et al*.^10^ on the same data for comparison. Table 4 shows the performance evaluation of the CLAM model on the validation and test sets and its comparison with multiclass variant of the MIL model performance on the same sets. This performance comparison declares an improvement of 4.41% and 4.83% in accuracy for validation set and test set, respectively, for CLAM over the MIL model.

**Table 4.** Performance evaluation of the CLAM and MIL models on validation and test sets.

Model	Valid set		Test set	
	Accuracy (ACC) (%)	Area under the ROC curve (AUC) (%)	ACC (%)	AUC (%)
CLAM	85.57	95.19	87.04	95.06
MIL	81.16	90.13	82.21	90.54

CLAM: constrained attention multiple instance learning; MIL: multiple instance learning.

**Figure 3.**
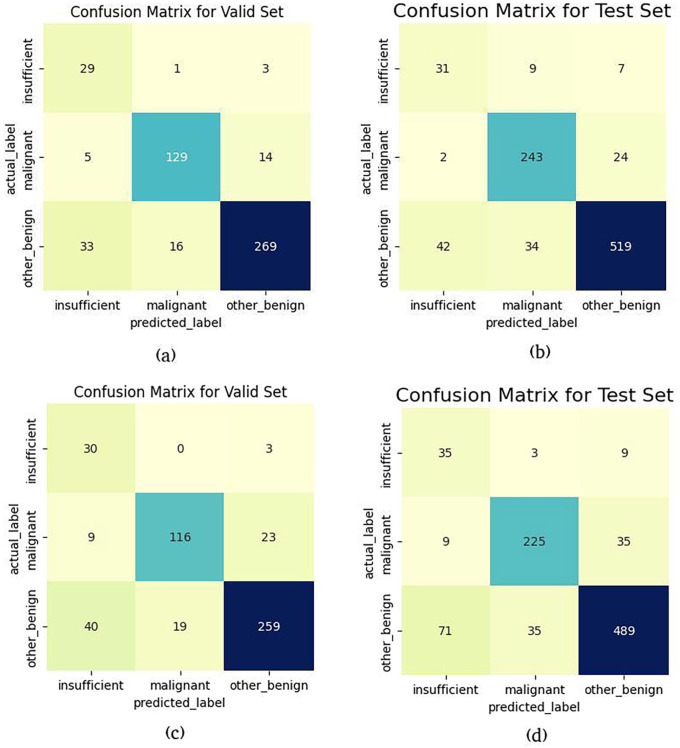
Confusion matrices for valid and test datasets (patch size, 256 × 256). (a) Confusion matrix for valid set (CLAM), (b) confusion matrix for test set (CLAM) (c) confusion matrix for valid set (MIL), and (d) confusion matrix for test set (MIL). (A color version of this figure is available in the online journal.)

Figure 3 shows the confusion matrices for validation and test sets for both models. Figure 3(a) and (b) shows the confusion matrices over validation and test sets for the CLAM model, and Figure 3(c) and (d) shows the confusion matrices over validation and test sets for the MIL model. As previously noted, the dataset used in this experiment exhibits significant class imbalance – the insufficient class has far fewer samples compared with the other classes. A malignant slide can contain both malignant and benign tissue, alongside blood and mucus. Insufficient slides contain very little tissue and some blood or mucus in most of the cases. Benign or Other labeled slides do not contain any malignancy but can also contain blood or mucus. This makes the separation between classes more difficult for the model to learn, particularly as far fewer insufficient labeled slides are available. The CLAM model does far better for recognizing the malignant slides than the MIL model. The sensitivity of the malignant class for the CLAM model is 87.16% and 90.33% over validation and test sets, while for the MIL model, the sensitivity of the malignant class is 78.33% and 83.64% over validation and test sets, respectively.

**Figure 4.**
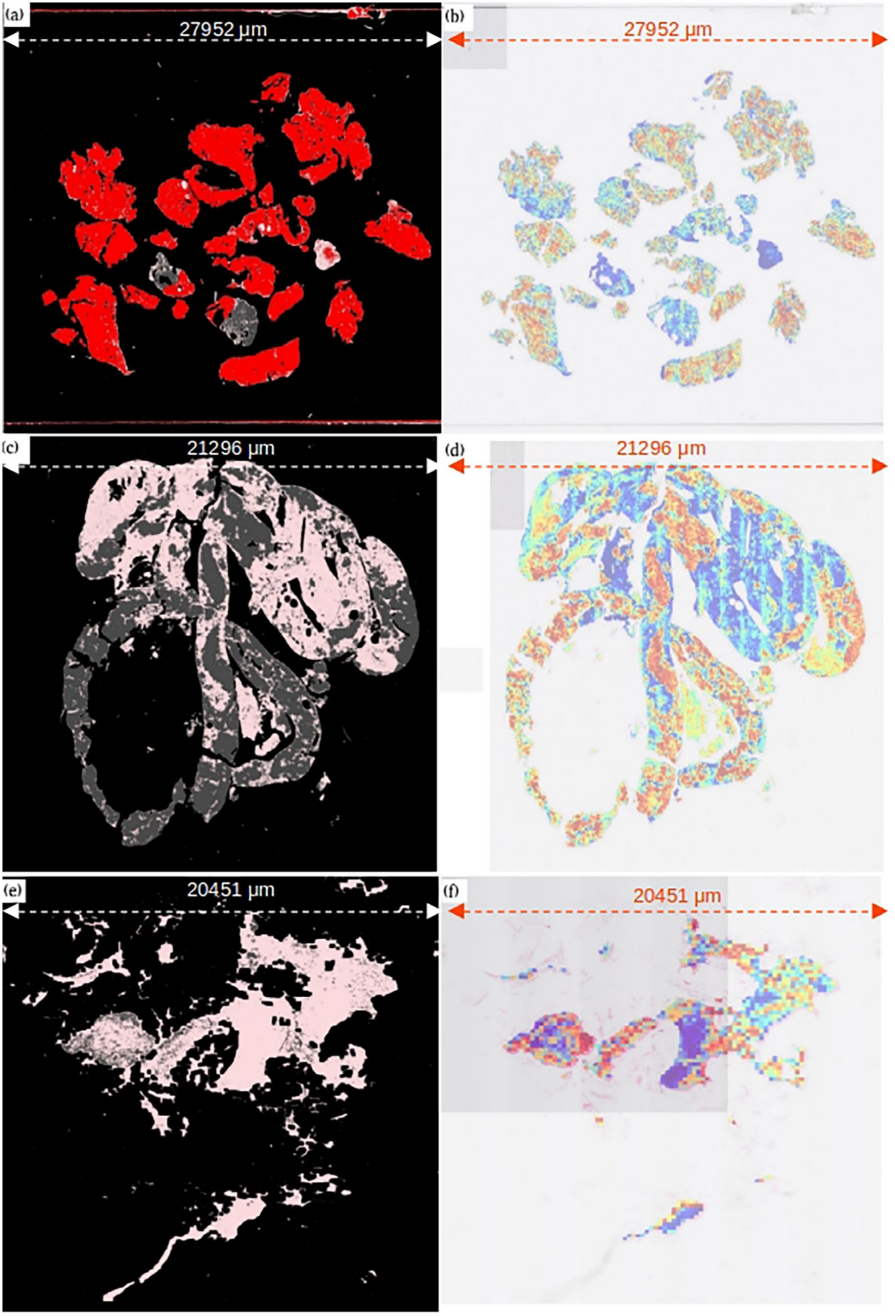
Comparison of predicted attention heatmaps with ground truth annotations provided by experts. (a) Malignant slide ground truth, (b) malignant slide–predicted attention heatmap, (c) normal/benign slide ground truth, (d) normal/benign slide–predicted attention heatmap, (e) insufficient slide ground truth, and (f) insufficient slide–predicted heatmap. For ground truth: Red: malignant tissue, gray: normal tissue, pink: blood/mucus, black: background (non-tissue). For attention heatmap: Red: highest attention regions, blue: lowest attention regions. (A color version of this figure is available in the online journal.)

Human-readable interpretability of CLAM models is easily available via patch-level attention heatmaps. These heatmaps identify the importance of different regions in a slide to the model’s final slide-level prediction as shown in Figure 4.

Examples of generated attention heatmaps for each of the slide-level categories serve high similarity between the strongly attended regions highlighted by the trained CLAM models and the annotations provided by the pathologists and can be used to validate that the predictive basis of the model aligns with well-known morphology used by pathologists for clinical diagnosis. Figure 4(a), (c), and (e) is the ground truth annotations provided for three of the slides from malignant, insufficient, and normal/benign categories. In ground truths, pink color represents blood/mucus, red shows malignant tissue, and gray shows normal tissue. Figure 4(b), (d), and (f) is the attention heatmaps generated for the same slides. In generated heatmaps, the regions of the tissue in the slide with high diagnostic relevance (higher attention score) are in warm color shades and the regions with lower diagnostic relevance (low attention score) are in cool color shades. Red shows the regions with the highest attention scores.

**Figure 5.**
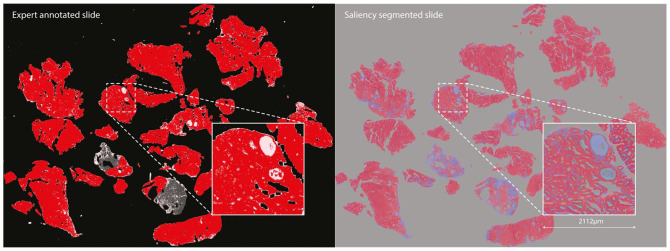
Comparison of areas identified as malignant (in red) by a human expert (left) and by our saliency-based segmentation method (right). (A color version of this figure is available in the online journal.)

**Figure 6.**
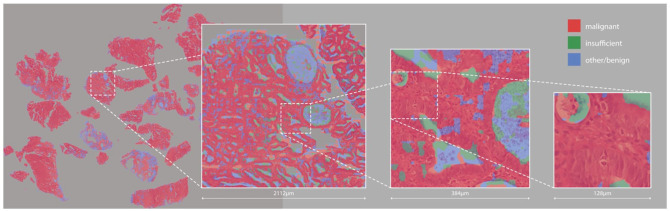
High-resolution saliency segmentation of a malignant slide. (A color version of this figure is available in the online journal.)

The fine-grained saliency segmentation maps (see Figure 6) identify similar regions to those labeled by experts, as shown in Figure 5, although with some interesting divergences, as we will discuss later.

Quantifying the results of these kinds of segmentation method is very time-consuming, as at high resolution the human expert annotations are actually far coarser than the saliency-based segmentation – so we do not really have access to a ground truth. Similarly, to our weakly supervised slides, any region of tissue may contain cells that are actually benign, and yet are still labeled malignant by humans, as visible in Figure 4. Human annotators do not work at the pixel level, and because of this we cannot directly compare the two. For this reason, we primarily consider the segmentation generated here as artifacts offering insight into the model’s behavior, as considered further in the “Discussion” section. However, for completeness and to evaluate how similar the saliency segmentation maps are with the ground truth segmentation provided by experts, we compared the saliency segmentation maps of correctly classified malignant WSIs in validation set (total 129 malignant WSIs) with their ground truth segmentation provided, using standard segmentation metrics as defined in the following equations. This comparison produced an average accuracy of 0.89 and an *F*1 score of 0.59, with 0.72 precision and 0.52 recall:

$$tp=\sum_{x,y=0}^{d}\tau_{x,y}\rho_{x,y}\;tn=\sum_{x,y=0}^{d}\left(1-\tau_{x,y}\right)\left(1-\rho_{x,y}\right)$$

$$fp=\sum_{x,y=0}^{d}\left(1-\tau_{x,y}\right)\rho_{x,y}\;fn=\sum_{x,y=0}^{d}\tau_{x,y}\left(1-\rho_{x,y}\right)$$

$$P=\frac{tp}{tp+fp}\;R=\frac{tp}{tp+fn}\;F1=\frac{2PR}{P+R}\;A=\frac{tp+tn}{tp+tn+fp+fn}$$

where *tp* is the true positive rate, *fp* is the false positive rate, *tn* is the true negative rate, and *fn* is false negative rate. *P* is precision, *R* is recall, *F*1 is *F*-score, and *A* is accuracy.

**Figure 7.**
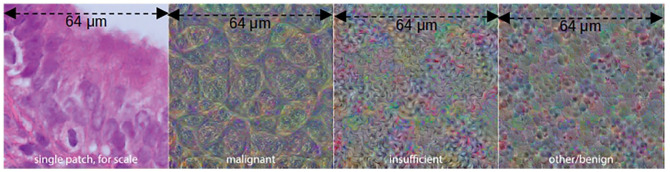
Input images optimized to maximize output logits for each class. (A color version of this figure is available in the online journal.)

To visualize the features for each class, we begin with a zero input matrix of size 256 × 256 (equal to the slide patch size) and optimize the pixels in it using gradient descent to maximize the output logit for each class in turn, using a learning rate of 0.001. We do this for 1000 epochs for each class, producing the feature visualizations shown in Figure 7.

## Discussion

Despite the promising results of automatic WSI processing for digital pathology using deep convolutional neural networks, the shortage of annotations has become the major bottleneck of WSI diagnosis model development.^18^ Manual annotation of gigapixel WSIs which is a requirement for these models is a laborious and time-consuming task. Weakly supervised learning models have gained popularity due to their ability to classify tissue without any need of detailed annotations.

Endometrial cancer is one of the most common types of gynecological cancer among women around the world.^19,20^ Early diagnosis of endometrial cancer types can help to save lives of the patients, and studies such as ours demonstrating the performance of weakly supervised learning methods on real-world data are key to enabling widespread adoption of these powerful techniques.

Our results indicate an overall good separation percentage between the categories for both validation and test sets, although the confusion matrices (shown in Figure 3) in section “Materials and methods” clearly show that there is some confusion between insufficient and normal classes and that the model has not been able to distinguish between these two categories well. This can be due to two reasons: first, the number of WSIs for the insufficient category is far lower compared to the other two categories; and second, the WSIs in this experiment can be multiclass (multi slide-level categories) as well as multilabel (i.e. a slide can contain tissue from more than one category). An insufficient WSI usually contains a tiny bit of normal tissue, and the rest of the WSI is blood or mucus – thus explaining the model’s confusion between these two categories. For practical purposes, accuracy in labeling malignant slides is key for obvious reasons – and for this class, our model performs well.

Classification accuracy is the most used metric for evaluating classification models. It can also be still a useful metric when the class distributions are slightly skewed. When the skew in the class distributions are severe, accuracy cannot be a reliable metric for measuring the performance.

Area under the receiving operating characteristic curve (AUC) is a useful tool for evaluating the quality of class separation for soft classifiers. In the multiclass setting, we can visualize the performance of multiclass models according to their one-versus-all precision-recall curves. AUC is also generalizable to multiclass problems. Table 4 in section “Results” shows that the AUC of both validation and test sets is higher than accuracies. This means that our model has been able to discern well when each class is measured against the others (AUC), but not as well when the prediction probabilities are an output of the softmax function, and therefore spread out for the three classes.

### Interpretability

In this section, we will consider the interpretability methods described in section “Materials and methods” and discuss them with the support of expert pathologists to better understand what our model has learned during training, and so gain insight into how to perform this classification task.

It is important to note that saliency-based segmentation maps are different in kind to attention heatmaps, although they may look similar. Attention heatmaps are generated at the patch level and show the *amount of attention given to each patch* by the attention backbone in order to produce the final, overall classification for that slide. In contrast, saliency-based segmentation maps work at the pixel level and show how *salient* each element of the original input image was for *each class*, in a completely model-agnostic fashion – we do not access the attention scores at all, and we calculate saliency with respect only to the relationship between the input image and each class logit.

**Figure 8.**
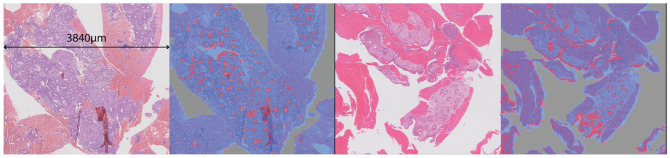
Tissue and saliency segmentations cropped from two benign slides – malignant saliency shown in red and benign in blue. Note the highly accurate epithelium segmentation associated with the malignant class: although the model correctly classified both slides as benign, it has learned that epithelium is highly salient for endometrial malignancy. (A color version of this figure is available in the online journal.)

In consultation with expert pathologists, we are able to make a number of observations from these detailed saliency maps and feature visualizations (examples shown in Figures 6 and 7). In both malignant and benign slides, the saliency maps show that the model finds epithelial structure (both glandular and surface) highly salient for the malignant class – whether the tissue is malignant or not. This is so pronounced that it could be mistaken for an epithelium segmentation model, particularly in benign slides where epithelial tissue is the only thing shown in red (see Figure 8). Endometrial cancer *is* an epithelial tumor and is typified by glandular abundance and complexity – so we are reassured by the fact that our model appears to have learned this. Note that while the model sees both benign and malignant epithelium as salient for malignancy at the pixel level, which may seem counter-intuitive, it is also very accurate in its slide-level classifications. This suggests that while the model uses the existence of epithelium in the slide as evidence of malignancy, it is also well able to distinguish between a normal arrangement of epithelium and an abnormal one. Pathologists do this by looking at the proportion of epithelium to stroma, and the architecture and complexity of glands present in the tissue – and based on the emphasis on epithelium and glands shown by the saliency maps, it seems possible that our model has learned to do something similar. This primacy of epithelium is supported by the malignant feature visualization shown in Figure 5. Our pathologists note that the patterns visible in this optimized input bear a strong resemblance to epithelial cells, which are round-ish, contain round nuclei, and are separated from each other by well-defined cell membranes. In any case, we now know that the model is highly unlikely to be making classifications based on some spurious correlation in the data, such as the amount of tissue or differences in staining.

The attention heatmaps and saliency segmentation maps generated show a high similarity between the prediction at patch level and pixel level with the ground truth segmentation provided by the pathologists.
